# Recurrence of Solid Pseudopapillary Tumor: A Rare Pancreatic Tumor

**DOI:** 10.1155/2016/7523742

**Published:** 2016-11-22

**Authors:** Chandra Punch, Nupur Garg, Penelope Harris

**Affiliations:** Madigan Army Medical Center, Tacoma, WA, USA

## Abstract

Solid pseudopapillary tumor of the pancreas (SPTP) is a rare disease of young females that does not usually recur after resection. Here we report a case of an elderly female with history of SPTP ten years ago who presented with anorexia and a palpable left lower quadrant abdominal mass. Imaging revealed metastatic disease and US-guided biopsy of the liver confirmed the diagnosis of SPTP. Due to her advanced age and comorbidities, she elected to undergo hospice care. The objective of this case report is to increase awareness of this tumor and its possibility of recurrence, necessitating further guidelines for follow-up.

## 1. Introduction

Solid pseudopapillary tumor of the pancreas (SPTP) is a rare pancreatic neoplasm described as a benign entity with occurrence most commonly seen in younger females (average age of 24 years) [1]. Most cases are diagnosed as an incidentally noted large mass; however when symptomatic it usually causes abdominal pain. The tumor is generally indolent in nature and survival following primary resection approaches 95% at 5 years with recurrence rates of 6.2% reported in case reports [2]. We describe a unique case of SPTP in an elderly female with recurrence many years after her primary diagnosis.

## 2. Case

**Figure 1:**
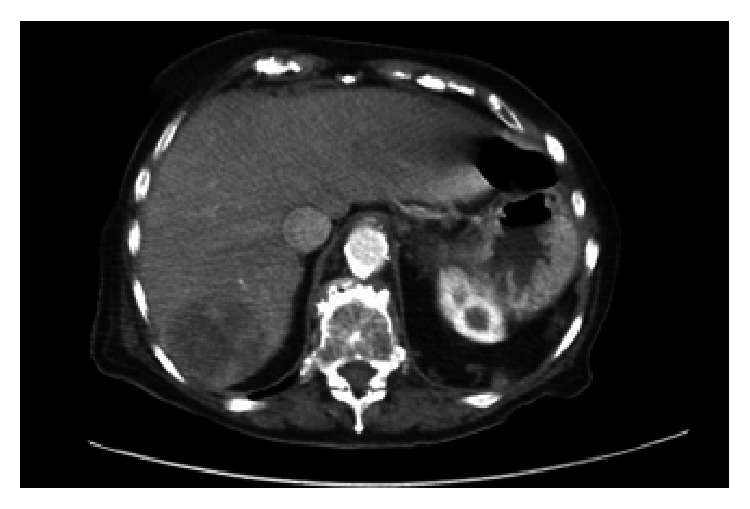
Axial view of abdominal CT scan with a hypodense mass in the liver measuring 50*∗*55 mm in size.

**Figure 2:**
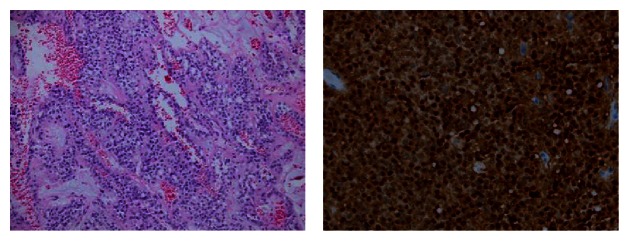
Pathology of solid pseudopapillary tumor of the pancreas H and E stain and B-catenin stain with the prominent nuclear staining.

An 88-year-old Caucasian female with history of SPTP diagnosed and resected 10 years prior to pathology revealing Ki-67 index of 2-3% presented with complaints of anorexia, dry heaving, increased somnolence, and a palpable left lower quadrant abdominal mass. Imaging revealed scattered soft tissue masses throughout the abdominal mesentery as well as multiple hypodense, heterogeneously enhancing lesions within the liver (Figure 1), consistent with metastases and similar to her imaging findings at diagnosis. US-guided biopsy of the liver confirmed the diagnosis of metastatic SPTP, staining positively for vimentin, CD10, and neuron-specific enolases (Figure 2). Due to her advanced age and comorbidities, she was a poor surgical candidate and elected best supportive care. She was transferred to inpatient hospice and passed away approximately a month after her initial presentation.

## 3. Discussion

Solid pseudopapillary tumor of the pancreas was first described by Dr. Virginia Frantz in 1959 and later the World Health Organization (WHO) in 1996 defined the tumor as a low grade malignant neoplasm of the exocrine pancreas [1]. The tumor currently represents only 1-2% of exocrine pancreatic tumors and has rarely been recorded in elderly females, such as our patient, initially diagnosed at 78 years old. Often an incidental finding, it can be associated with abdominal discomfort/pain, a palpable mass, anorexia, nausea, vomiting, and weight loss [1]. The etiology of SPTP is unknown; however it has been postulated to derive from either primitive pancreatic cells or cell lines of the female genital bud [3]. Imaging (US, CT/MRI) is the primary diagnostic tool and will reveal a solid and cystic mass with irregular calcifications seen in 20% of cases [4]. Definitive diagnosis is confirmed with needle biopsy. Cytologic analysis is diagnostic in 75% of cases with positive vimentin, CD10, and beta-catenin stains differentiating SPTP from pancreatic neuroendocrine tumors. Histology will often reveal branching papillae with myxoid stroma [5]. This tumor has been reported to reach up to 18 cm and even then is generally thought to be of low malignant potential and overall excellent prognosis. Malignant cases are defined as solid pseudopapillary carcinomas and are treated with resection [6]. Metastases are seen in 10–15% of advanced cases, with a tendency to localize to regional lymph nodes, liver, and peritoneum/omentum. Additional treatments, including chemotherapy and radiotherapy, have been noted in some cases with good response but are in general not used, and the 5-year survival rate after resection approaches 95% [7].

## 4. Conclusion

While previously described as a tumor of young females, solid pseudopapillary tumor of the pancreas (SPTP) can occur in advanced age. Although the five-year survival rate following resection approaches 95%, tumor recurrence has been reported, potentiating a role for follow-up guidelines.
